# Wide Complex Irregular Rhythm in a Paced Patient: A Clinical Approach

**DOI:** 10.3390/reports8030109

**Published:** 2025-07-16

**Authors:** Haralambie Macovei, Andrei Mihordea, Cristina Andreea Adam, Lucia Corina Dima-Cozma, Elena-Andreea Moales, Maria-Magdalena Leon, Florin Mitu

**Affiliations:** 1 Clinical Rehabilitation Hospital, Cardiovascular Rehabilitation Clinic, Pantelimon Halipa Street No. 14, 700661 Iasi, Romania; 2 Department of Medical Specialties I, “Grigore T. Popa” University of Medicine and Pharmacy, University Street No. 16, 700115 Iasi, Romania

**Keywords:** pacemaker malfunction, atrial fibrillation, Holter, ECG, heart conduction system disease, device failure, accidental falls, elderly patient, cardiac arrhythmias

## Abstract

**Background and Clinical Significance:** Evaluating wide complex rhythms in patients with permanent pacemakers can be a diagnostic challenge, particularly when the rhythm is irregular. While pacemaker-mediated rhythms are typically regular and predictable, the appearance of wide complex irregular rhythms raises concerns ranging from lead malfunction to life-threatening arrhythmias, such as ventricular tachycardia. Understanding the interplay between intrinsic cardiac activity and device function is crucial for timely and accurate diagnosis in this increasingly common clinical scenario. **Case presentation:** We report on a 74-year-old female with a VVI pacemaker implanted for binodal disease, who presented with intermittent palpitations and an irregular rhythm. The patient has a recent history of falling on her right shoulder, which is also the site of the device implantation. We used a clinical step-by-step approach to rule out pacemaker malfunction and to establish the need for an unscheduled device interrogation. **Conclusions:** This case presentation highlights the important role of clinical reasoning and the approach to such a patient, especially when a key method of pacemaker evaluation, such as device interrogation, is not readily available.

## 1. Introduction and Clinical Significance

The effective functioning of the heart is reliant on the cardiac excitoconductory system, which comprises impulse generators, such as the sinoatrial node, and the impulse-propagating system known as the His-Purkinje system [1,2]. Various pathological conditions can impact the conduction system, influencing either impulse initiation or propagation, or both, generating arrhythmias. Common acquired causes of conduction system dysfunction include myocardial infarction, age-related degenerative changes, complications arising from invasive procedures, and drug-induced toxicity [3].

The current standard of care for managing symptomatic bradyarrhythmia involves a cardiac implantable electronic device (CIED). These pacing devices deliver controlled electrical impulses to facilitate myocardial cell depolarization, thereby helping to maintain the electrical excitability of cardiac tissues [4]. Despite their effectiveness, electronic pacemakers are accompanied by several limitations, such as procedural risks associated with implantation, finite battery lifespan, potential for infections, and risks of device malfunction [5].

Evaluating wide complex rhythms in patients with permanent pacemakers represents a significant diagnostic challenge, particularly in instances of irregular rhythms. Typically, rhythms facilitated by pacemakers are characterized by their regularity and predictability, which offer a sense of assurance to both clinicians and patients. However, the manifestation of wide complex irregular rhythms raises concerns, from potential lead malfunction to life-threatening arrhythmias, such as ventricular tachycardia [6]. A thorough understanding of the interplay between intrinsic cardiac activity and CIED function is essential for timely and accurate diagnosis in this increasingly common clinical scenario.

Clinical Significance: This case presentation emphasizes the critical role of clinical reasoning and a systematic approach when managing such patients, particularly in situations where a fundamental method of pacemaker evaluation, such as device interrogation, is not readily available. Recognizing these complexities is vital for ensuring high-quality patient care.

## 2. Case Presentation

A 74-year-old female presented with intermittent palpitations characterized by an irregular rhythm. She had an initial pacemaker implantation 12 years ago for binodal disease and had undergone an elective pulse generator replacement with a St. Jude Edurity single-chamber pacemaker two years ago. In recent weeks, she had experienced poor balance, resulting in multiple falls onto her right shoulder, the site of her pacemaker implantation. Additionally, she described exertional dyspnea during moderate physical activity, such as gardening.

During the physical exam, the patient was stable both hemodynamically and respiratorily, with no evidence of acute distress. Inspection of the pacemaker pocket revealed no abnormalities, and the initial assessment was negative for overt traumatic injury, pneumothorax, or pleural or pericardial effusion.

**Figure 1:**
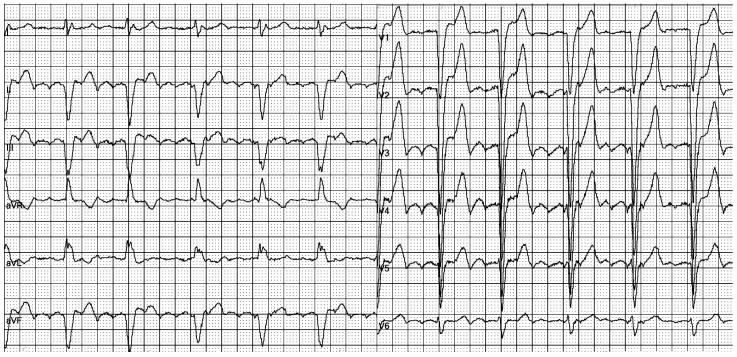
**12-lead ECG of our patient at admission**: There is an irregular rhythm with wide complex QRS and a left bundle branch morphology. The average heart rate is 75 bpm.

The electrocardiogram (ECG) revealed an irregular rhythm with broad QRS complexes (Figure 1), leading to her admission with a suspicion of pacemaker malfunction.

**Figure 2:**
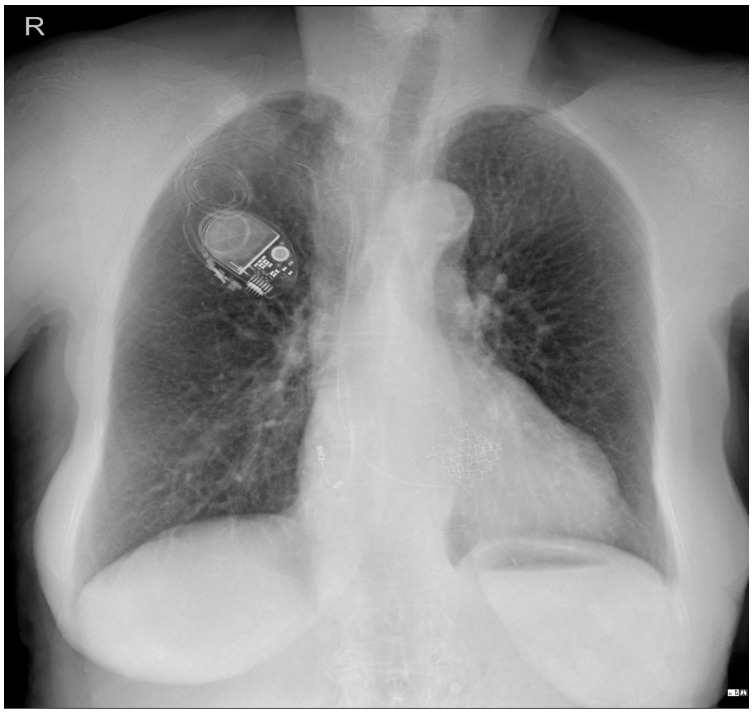
**Patient chest X-ray at admission**: a cardiac pacemaker is present in the right pectoral region with a ventricular stimulation lead placed on the free wall of the right ventricle and an abandoned atrial lead. There is no apparent lead displacement or fracture. The inferior segment of the left border of the heart is elongated and bulging. An aortic prosthetic valve is present. There are no pleural or pulmonary lesions.

**Figure 3:**
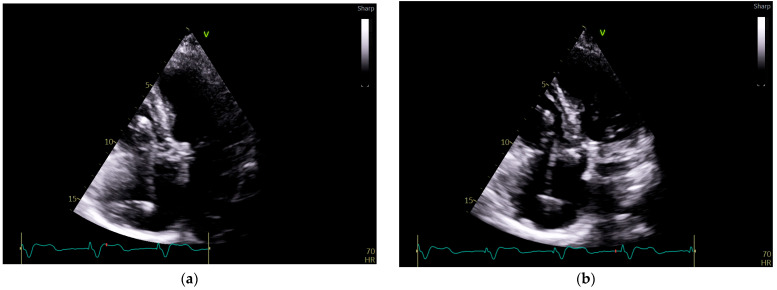

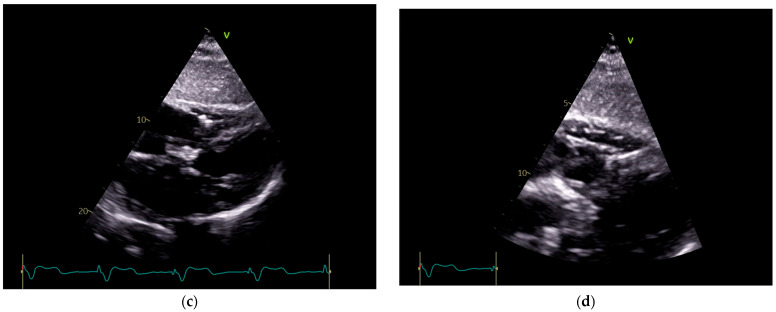
**Echocardiography exam images at admission:** A transthoracic echocardiography (TTE) exam was performed in the apical 4-chamber view (**a**,**b**) and subcostal view (**c**,**d**), revealing the insertion of the pacing probe into the myocardium of the right ventricle free wall, without any visible fractures or ruptures of the lead.

To investigate a potential cause of malfunction of the intracardiac device, it was decided that both a chest X-ray (Figure 2) and an echocardiogram (Figure 3) would be performed. These diagnostic tests were selected to confirm or rule out the possibility of a pacing probe fracture, particularly considering the patient’s recent fall, which may have compromised the integrity of the device or the intracardiac pacing leads. The chest X-ray assesses the device positioning and identifies potential fractures of the pacing leads. Concurrently, the echocardiogram provides critical information regarding cardiac function and its interaction with the implanted device. Together, these diagnostic modalities are essential for optimal management of the patient.

Given that the patient was stable and in no acute distress, we conducted Holter monitoring to observe any potential rhythm disturbances, before referring her for electrophysiologic consult.

**Figure 4:**
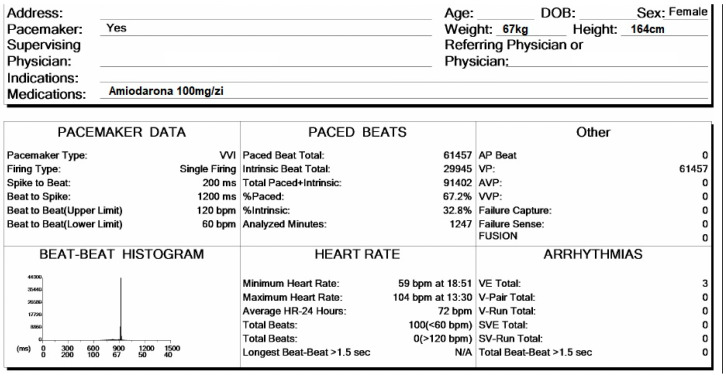
Holter ECG monitoring summary, presenting the general data of the patient and heart rhythm parameters.

In Figure 4, the patient’s general data are presented. She is a 74-year-old female, weighing 67 kg and standing 164 cm tall. She is currently on a daily dosage of 100 mg of Amiodarone. The presence of a pacemaker has been checked to identify stimulation spikes. During the 20 h and 47 min of monitoring, a total of 91,402 beats were recorded, of which 67.2% were paced beats. The average heart rate was 72 bpm, with a minimum of 59 bpm and a maximum of 104 bpm.

**Figure 5:**
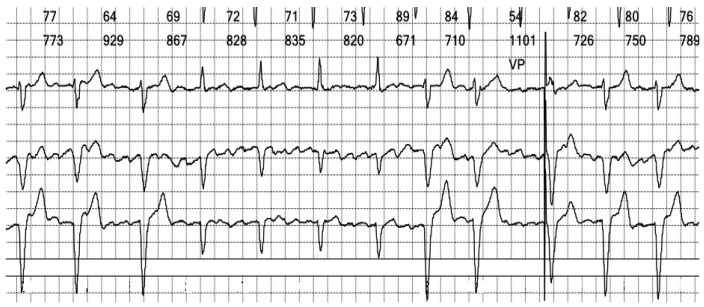
**Holter ECG strip**: Wide QRS beats alternate with narrow complexes in an irregular sequence. Wide QRS beats have approximately the same morphology, but only one is preceded by a stimulation spike. Between the QRS complexes, the atrial activity can be observed, replacing the isoelectric line.

**Figure 6:**
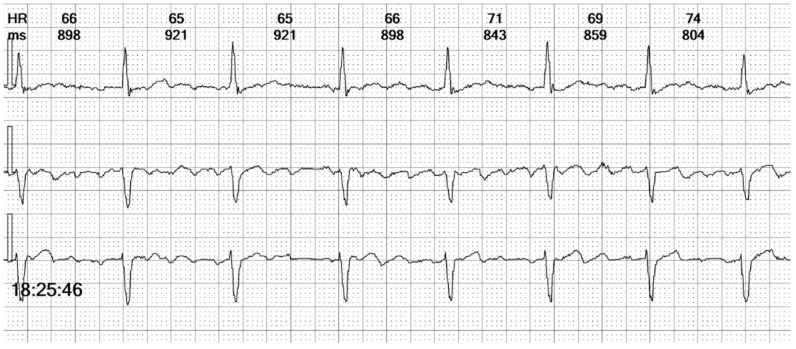
**Holter ECG strip**: Atrial activity seems disorganized, suggestive of coarse wave atrial fibrillation (see Figure 11A). This pattern is frequent in patients under Amiodarone. The intrinsic rhythm exhibits a narrow complex QRS, reflecting the fast activation through the specialized conduction system (see Figure 13A).

**Figure 7:**
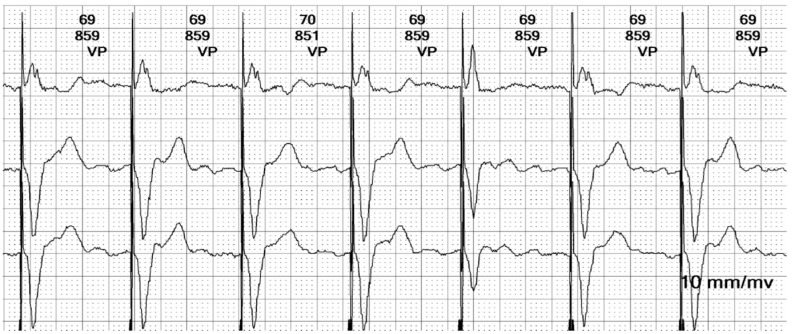
**Holter ECG strip**: The ventricular stimulated rhythm has a rate of 70 bpm. The pacemaker-generated spike is followed by a wide QRS complex. The lead is placed at the apex of the right ventricle (see Figure 3 and Figure 13C), depolarizing the right ventricle before the left ventricle. The lead is in contact with the myocardium, and the conduction is slow, cell-to-cell, thus generating a broad QRS, reflecting the interventricular and intraventricular dyssynchrony.

**Figure 8:**
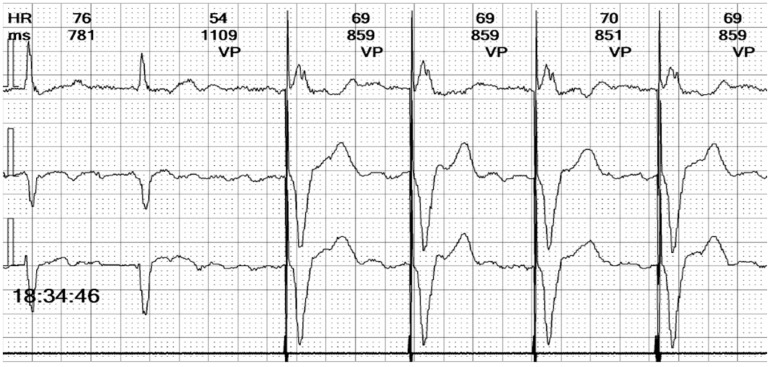
**Holter ECG strip**: Hysteresis: The paced rhythm is set at 70 bpm, but pacing is triggered at a lower heart rate, of 55 bpm. This demonstrates that the hysteresis option is activated, to favor the intrinsic rhythm rather than the right ventricle pacing that induces dyssynchrony.

**Figure 9:**
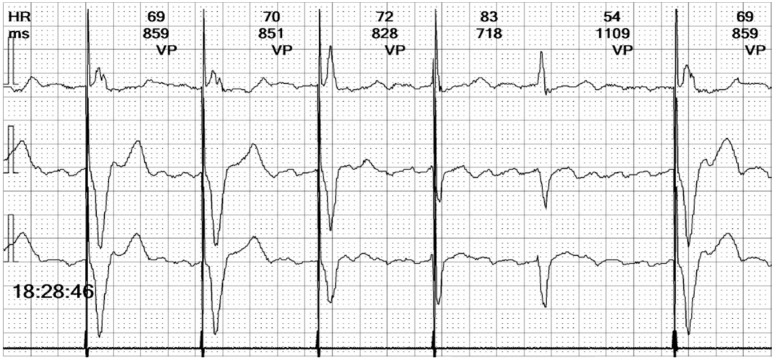
**Holter ECG strip**: Paced beat morphologies: Fusion (beat 3), pseudofusion (beat 4), intrinsic beat (beat 5), and stimulated complexes (beats 1, 2, and 6). The fusion beat has an intermediary pattern between the stimulated and intrinsic beat, due to the electrical activation through both the conduction system and the pacemaker lead (see Figure 13D). The pseudofusion has the same pattern of an intrinsic beat with a spike overlayed; the spike is delivered in the absolute refractory period and does not depolarize the myocardium (see Figure 13E).

**Figure 10:**
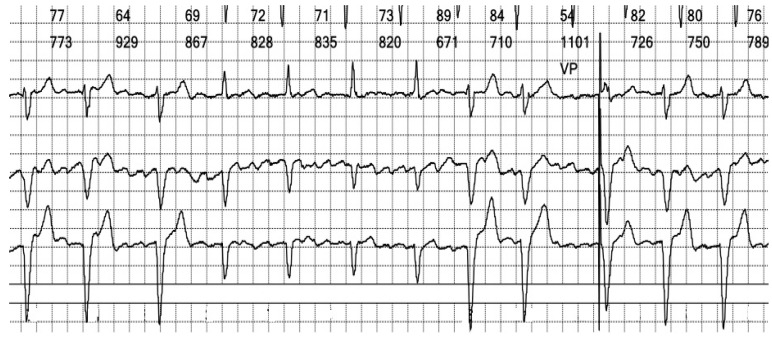
**Holter ECG strip**: Intermittent bundle branch block: At frequencies above 75 bpm, the QRS becomes wide and there are ST-T changes. It should be noted that the rhythm is irregular, the heart rate is above the pacing rate, and there is no spike, meaning that the beats are not stimulated but conducted with a frequency-dependent bundle branch block.

Figure 5, Figure 6, Figure 7, Figure 8, Figure 9 and Figure 10 show the most relevant ECGs recorded by the three-channel Holter monitor.

In summary, the Holter monitor revealed a pacemaker rhythm that alternates with periods of spontaneous conduction, all occurring against a background of continuous atrial fibrillation (Figure 5 and Figure 6). The mean ventricular rate is 72 beats per minute (bpm). The ventricular stimulation (Figure 7 and Figure 8), which accounts for 67% of the recorded time, generates a wide QRS complex and has a rate of 70 bpm, triggered at intervals longer than 1090 milliseconds (ms) when the heart rate is below 55 bpm. A few fusion and pseudofusion beats were noted (Figure 9); however, there were no sensing or capture failures observed. The spontaneous conduction shows a heart rate ranging from 55 bpm to 104 bpm, with intermittent left bundle branch block seen at ventricular rates greater than 75 bpm (Figure 10). No ventricular arrhythmias were recorded during the monitoring period.

**Figure 11:**
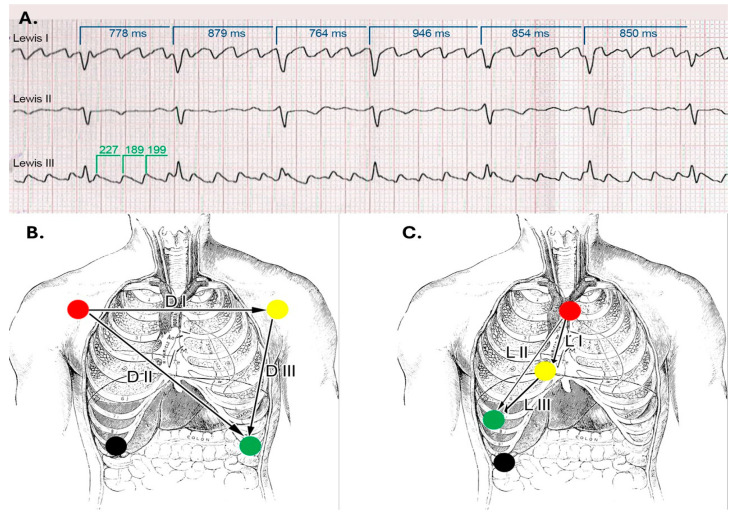
**Lewis Leads:** (**A**). ECG strip showing the atrial waves, which are slightly polymorphic and with different frequencies. The ventricular rate is variable, suggesting that the atrial rhythm is indeed atrial fibrillation with coarse waves. (**B**). Lead placement in standard and (**C**). Lewis configurations. (Figure 11B,C are adapted from a public domain image).

**Figure 12:**
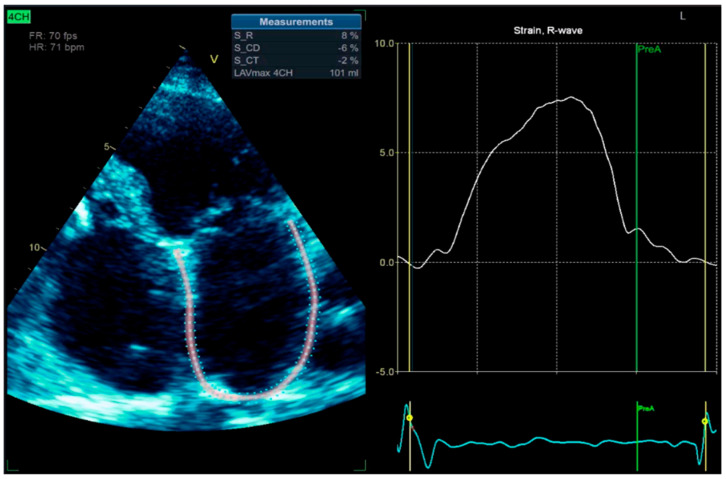
2D-TTE speckle-tracking strain imaging shows enlargement of the left atrium with an increase in the left atrial volume index (LAVi = 101 mL), demonstrating a loss of contractile phase (−2%) and atrial stunning due to atrial fibrillation.

To enhance atrial activity recording, a modified surface ECG was performed using the Lewis lead placement. The Lewis lead is a modified ECG lead configuration designed to enhance the detection of atrial activity, which is particularly useful in identifying P waves during arrhythmias such as atrial flutter or atrial tachycardia [7]. In this setup, the right-arm electrode is placed on the manubrium of the sternum, and the left-arm electrode is placed on the right fifth intercostal space close to the sternum, with the left-leg electrode remaining in its standard position. This orientation essentially aligns the lead to the atrial vector, providing a clearer view of atrial depolarization (Figure 11) [8]. We also performed left atrium strain by the speckle-tracking echocardiography technique, which confirmed atrial stunning with a loss of contraction (LA contraction strain was −2%), a specific change in atrial fibrillation (Figure 12).

**Clinical Approach**: Pacemaker malfunction was ruled out, and the patient’s wide complex irregular rhythm was determined to be due to atrial fibrillation, with intermittent left bundle branch block. Based on the modified European Heart Rhythm Association (EHRA) symptom classification, we categorized her condition as symptomatic atrial fibrillation, mEHRA Class 3 [9]. Amiodarone was substituted with a beta-blocker to achieve better rate control, and the patient was continued on anticoagulation therapy. After a couple of days, the patient described the resolution of fast rhythm palpitations. She was instructed to follow her regular pacemaker check-ups.

## 3. Discussion

Pacemaker-related late complications, defined as those occurring more than three months after device implantation, have been reported in approximately 0.5% to 7.5% of patients [10], with an incidence of around 1.4% following initial single-chamber pacemaker implantation [11]. This incidence increases after generator replacement, primarily due to a higher rate of pocket erosions [11]. It is noteworthy that the incidence of pacemaker-related complications has declined over time. For example, one study reported a reduction in the annual pacemaker malfunction replacement rate from 0.9% in 1993 to 0.14% in 2002 [12].

Late complications in paced patients may be hardware-associated, involving the leads—such as lead fracture, insulation defects, lead-generator connector issues, or delayed lead perforation—or may be due to the pulse generator itself, including premature generator failure, Twiddler’s syndrome, pocket erosion, or infection [10].

In the event of a suspected malfunction of a pacemaker in a stable patient, the initial assessment should start with a surface ECG and a chest radiography, which are essential in evaluating the pacemaker’s hardware and function. Chest radiography is effective in identifying lead displacement and gross lead fracture, although the latter is uncommon with modern devices. Chest X-ray alone has 71% sensitivity and 100% specificity in diagnosing lead fracture [13].

In the elderly population, myocardial perforation by the pacing lead has an incidence of 2.2% [14]. Chest radiography has a sensitivity of 27.7% and a specificity of 94.4% in identifying this complication. The addition of echocardiography is valuable in this setting, as it has an increased diagnostic sensitivity of 41.2%, while the specificity is 84.2% [15]. However, chest radiography is limited in its ability to detect subtle abnormalities, such as lead insulation defects or microdisplacement of the pacing electrode [16].

Besides mechanical complications, pacemaker functional anomalies must be taken into account. These include sensing issues (undersensing and oversensing due to far-field sensing, T-wave sensing, or noise) or pacing problems (loss of capture, failure to pace, battery depletion) [10]. Surface ECG and Holter monitoring have high sensitivity and nearly 100% specificity for diagnosing these malfunctions. A systematic analysis of the tracings can suggest the probable malfunction or arrhythmia [17]. Nevertheless, if ECG or Holter indicate pacemaker malfunction, device interrogation is mandatory in order to confirm and address the problem. As such, ECG findings can be correlated with the intracavitary electrograms and device parameters.

In the event of pacemaker malfunction, early identification is crucial to ensure timely intervention. Severe adverse outcomes are uncommon but should not be underestimated. In a cohort of 2.25 million patients with pacemaker implants observed from 1990 to 2002, 8834 (0.39%) pacemakers had to be explanted, and 30 deaths (1.3 deaths per 100,000 patients) were attributed to device malfunction [18].

Pacemaker recipients have an increased prevalence of atrial fibrillation in comparison to other patient groups, with a prevalence as high as 30–40% [19]. Atrial fibrillation is especially prevalent in patients with VVI pacing and sick sinus syndrome [20]. Several mechanisms have been proposed to explain this association, including atrioventricular dyssynchrony, atrial retrograde depolarization, mitral and tricuspid regurgitation, increased atrial pressure, and pulmonary vein distension. These hemodynamic and electrophysiological alterations are believed to beget atrial structural and electrical remodeling [21], ultimately leading to atrial cardiomyopathy and atrial fibrillation. It should be noted that VVI pacing has a high rate of ventriculo-atrial retrograde depolarization (80% in sick sinus syndrome versus 35% in AV block patients [22]), leading to atrioventricular dyssynchrony, atrial contraction with a closed mitral valve, increased atrial pressure, and flow reversal in the pulmonary veins, promoting atrial fibrillation [23].

The onset of atrial fibrillation in a paced patient raises some difficulties in interpreting the ECG, particularly when it overlaps with other electrical disturbances, for instance, binodal disease which develops into permanent atrial fibrillation. In these cases, the presence of slow atrial fibrillation or high-grade intermittent atrioventricular block can prolong pacing periods. An illustration of this was seen in our patient, where ventricular pacing was 67% of the recorded time, masking the heart’s spontaneous atrial rhythm.

Additionally, the challenge of interpreting the ECG intensifies with the presence of intermittent bundle branch block, especially the left bundle branch block (LBBB). Around 5% of patients with LBBB also have atrial fibrillation [24], and this comorbid association brings a higher risk of heart failure [25]. LBBB pattern can mimic the appearance of right ventricular pacing, and differentiating between the two is demanding when examining a three-channel Holter strip. In our patient’s case, the paced QRS complexes revealed a distinctive initial positive upstroke on Channel 1, equivalent to lead I (see the beat marked “VP” in Figure 10).

Intermittent bundle branch block (BBB) results from injury to the cardiac conduction system and may manifest with or without frequency dependence. It reflects the underlying pathology of the bundle branches, commonly due to fibrosis, ischemia, or degenerative changes. This phenomenon is particularly prevalent among elderly patients and those with preexisting conduction system disease [26]. In contrast, aberrant conduction arises from physiological differences in repolarization times between the bundle branches, leading to asynchronous ventricular depolarization. Aberrant conduction is generally considered a benign phenomenon and does not reflect intrinsic disease of the conduction system [27].

In our patient, we attribute the conduction disturbance to intermittent left bundle branch block rather than aberrant conduction. Several factors support this conclusion. First, the patient had a known diseased atrioventricular node, with a prior diagnosis of binodal disease; the appearance of bundle branch block is indicative of progressive conduction system degeneration. Second, the conduction abnormality predominantly manifested at heart rates exceeding 75 beats per minute, although it was occasionally observed at lower rates, suggesting that the mechanism was not exclusively rate-dependent. Finally, aberrant conduction typically involves the right bundle branch due to its physiologically longer repolarization time and is often precipitated by a short-long-short RR interval sequence, none of which were evident in this case.

**Figure 13:**
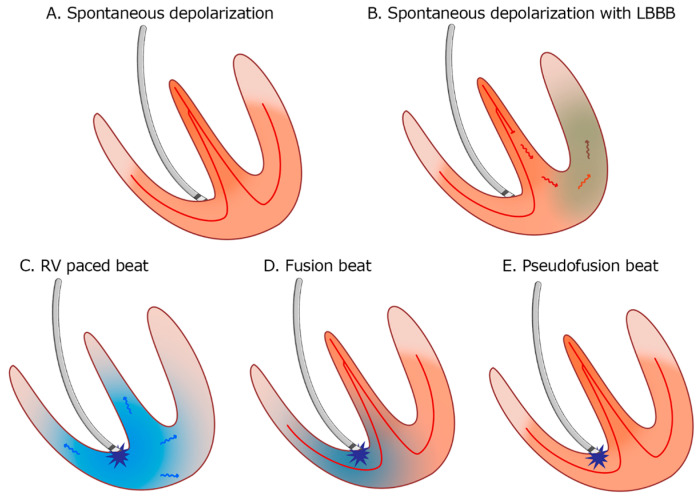
**Patterns of myocardial depolarization:** (**A**). Normal spontaneous depolarization through the conduction system. (**B**). Spontaneous depolarization with left bundle branch block (LBBB). (**C**). Right ventricle (RV) apical pacing. (**D**). Fusion beat—the depolarization occurs partially through the conduction system and partially by the pacemaker stimulus. (**E**). Pseudofusion—the myocardium is depolarized spontaneously only through the conduction system; the pacemaker stimulus is delivered in the absolute refractory period and does not activate the myocardium.

The occurrence of fusion and pseudofusion beats (see Figure 13) is not uncommon in paced patients, and it is a benign finding. It can be misinterpreted as a marker of inappropriate sensing or capture issues [28,29]. It is crucial to utilize the available techniques to distinguish atrial rhythm disorders effectively. For instance, employing Lewis right leads can provide a more accurate capture of right atrial activity compared to the conventional 12-lead ECG. Moreover, we should not overlook the value of echocardiographic imaging techniques, such as left atrial strain speckle-tracking. Left atrial (LA) strain imaging, derived from speckle-tracking echocardiography (STE), is a valuable non-invasive tool for assessing atrial function by quantifying atrial deformation during different phases of the cardiac cycle. LA strain, particularly during the reservoir phase, has emerged as a sensitive marker of atrial myocardial function and structural remodeling [30,31].

In the context of atrial tachyarrhythmias (ATAs), LA strain is particularly helpful in distinguishing atrial fibrillation from other organized ATAs, such as atrial flutter or atrial tachycardia. Patients with atrial fibrillation typically exhibit significantly reduced LA reservoir and conduit phase, with the absence of contraction phase and reduced global strain values compared to those with other ATAs, reflecting a greater degree of atrial fibrosis and disorganization of the atrial activity. These changes result in impaired LA compliance and function, which can be objectively captured using strain imaging [32,33].

Our clinical approach to suspected pacemaker malfunction is appropriate only for hemodynamically stable patients without suspicion of life-threatening arrhythmia and in settings lacking access to device interrogation hardware or specialized personnel. In the event of a paced patient who develops wide complex tachycardia, the first step is to reprogram the pacemaker to an asynchronous pacing mode (VOO or DOO), either by applying a magnet or through device interrogation, thereby terminating a potential device-related tachyarrhythmia (6% of all pacemaker recipients have at least one documented pacemaker-mediated tachycardia [22]). More commonly, however, pacemaker recipients develop wide complex tachycardias unrelated to the cardiac implantable electronic device (CIED). In hemodynamically stable patients, device interrogation provides intracardiac electrograms that can assist in differentiating arrhythmia.

If the patient is hemodynamically unstable and ventricular tachycardia or ventricular fibrillation is confirmed on the surface electrocardiogram, advanced cardiac life support protocols should be initiated without delay. External defibrillation is considered safe in patients with CIEDs, provided that defibrillator pads are positioned at a distance from the pulse generator (at least 8 cm) to avoid device damage [34].

## 4. Conclusions

This case highlights the diagnostic challenge associated with wide complex irregular rhythm in patients with pacemakers, atrial fibrillation, and conduction abnormalities, particularly when device malfunction is suspected. Confirming appropriate pacemaker function is essential for targeted therapeutic interventions; however, this process is sometimes complicated by limited access to device interrogation. In this case, simple methods were utilized to clarify the underlying rhythm. Although these methods may facilitate quick clinical decisions, further studies are needed to validate their accuracy and safety prior to their routine clinical implementation.

## Data Availability

The data presented in this study are available upon request from the corresponding author (ethical restrictions).

## Abbreviations

The following abbreviations are used in this manuscript:

ATAs	Atrial tachyarrhythmias
AVN	Atrioventricular node
bpm	Beats per minute
CIED	Cardiac implantable electronic device
ECG	Electrocardiography
LA	Left atrium
LBBB	Left bundle branch block
ms	Milliseconds
RV	Right ventricle
SAN	Sinoatrial node
STE	Speckle-tracking echocardiography
TTE	Trans-thoracic echocardiography
